# The selective expression of carbonic anhydrase genes of *Aspergillus nidulans* in response to changes in mineral nutrition and CO_2_ concentration

**DOI:** 10.1002/mbo3.311

**Published:** 2015-11-09

**Authors:** Leilei Xiao, Bin Lian, Cuiling Dong, Fanghua Liu

**Affiliations:** ^1^ Jiangsu Key Laboratory for Microbes and Functional GenomicsJiangsu Engineering and Technology Research Center for MicrobiologyCollege of Life SciencesNanjing Normal UniversityNanjing210023China; ^2^ Key Laboratory of Coastal Environmental Processes and Ecological RemediationYantai Institute of Coastal Zone ResearchChinese Academy of SciencesYantai264003China

**Keywords:** *Aspergillus nidulans*, carbonic anhydrase, CO_2_ adaption, isoenzyme, mineral weathering

## Abstract

Carbonic anhydrase (CA) plays an important role in the formation and evolution of life. However, to our knowledge, there has been no report on CA isoenzyme function differentiation in fungi. Two different CA gene sequences in *Aspergillus nidulans* with clear genetic background provide us a favorable basis for studying function differentiation of CA isoenzymes. Heterologously expressed CA1 was used to test its weathering ability on silicate minerals and real‐time quantitative PCR was used to detect expression of the CA1 and CA2 genes at different CO_2_ concentrations and in the presence of different potassium sources. The northern blot method was applied to confirm the result of CA1 gene expression. Heterologously expressed CA1 significantly promoted dissolution of biotite and wollastonite, and CA1 gene expression increased significantly in response to soluble K‐deficiency. The northern blot test further showed that CA1 participated in K‐feldspar weathering. In addition, the results showed that CA2 was primary involved in adapting to CO_2_ concentration change. Taken together, *A. nidulans* can choose different CA to meet their survival needs, which imply that some environmental microbes have evolved different CAs to adapt to changes in CO_2_ concentration and acquire mineral nutrition so that they can better adapt to environmental changes. Inversely, their adaption may impact mineral weathering and/or CO_2_ concentration, and even global change.

## Introduction

Carbonic anhydrase (CA) catalyzes the reversible hydration of carbon dioxide: ${\text{CO}}_{2}+H_{2}O↔H^{+}+{\text{HCO}}_{3}^{-}$ (Meldrum and Roughton 1933; Tripp et al. 2001). This ancient enzyme is widely distributed in various organisms, which implies that it played an important role in the process of the carbon biogeochemical cycle and the evolution of life (Kaur et al. 2012). CA was first found in mammalian erythrocytes (Meldrum and Roughton 1933), and almost all mammals have been found to have several CA isozymes (Kaur et al. 2012). Animal CA exists in different tissues and plays an important part in a variety of physiological processes such as: bone formation, calcification, ion transportation, acid‐base balance maintenance, and acid‐base transportation (Gilmour and Perry 2009). It is also important for the photosynthesis of many green plants (Badger and Price 1994; Badger 2003) and algae (Badger et al. 2002; Moroney et al. 2004) because of its catalytic action which provides sufficient bicarbonate (Wang et al. 2011). It suggested that CAs are related to CO_2_ utilization during photosynthesis. Photosynthetic microorganisms are also able to effectively utilize CO_2_ in lower concentrations through CA catalysis (Kaplan and Reinhold 1999). In addition, CA plays a key role in life processes (Smith and Ferry, 2000; Xiao et al. 2012) and CO_2_ fixation (Moroney et al. 2004; Barbero et al. 2013) in nonphotosynthetic microorganisms. The loss of CA activity could lead to growth defects and adversely affect other biochemical reactions involving substrates such as HCO_3_^−^ or CO_2_ (Kaur et al. 2012). To date, five independent evolutionary gene families (denoted by *α*,* β*,* γ*,* δ*, and *ζ*) have been found (Hewett‐Emmett and Tashian 1996; Tripp et al. 2001). The high level of overall similarity (97%) of two CA genes (CA1 and CA2) presented in *Chlamydomonas reinhardtii* (Villand et al. 1997), suggested that there were no functional differences between CA1 and CA2 combined with the observation that both genes were expressed at low concentrations of CO_2_. However, Kaur et al. (2012) questioned the significance of the co‐existence of several types of CA in the same species. Eriksson et al. (1996) showed that mitochondrial CA (mtCA) and periplasmic CA (pCA) probably have two distinct roles in *C. reinhardtii* cells: mtCA may be important for buffering the mitochondrial matrix and pCA supplies CO_2_ to the plasma membrane. The fact that many microbial genomes have more than one CA gene indicates its importance. When hostile environments threaten the survival of organisms, they strive to reduce the influence of adverse effects through gene evolution (Kis‐Papo et al. 2003; Romero et al. 2012) or regulation of the metabolic network by changing the expression levels of particular genes (Olson 2006). CO_2_ sensing and metabolism, with the help of CAs, play important roles in the proliferation, survival, and differentiation of diverse pathogenic fungi which infect human hosts (Tobal and Balieiro 2014). *β*‐carbonic anhydrase, which can be encoded by the CAN2 gene in *Cryptococcus neoformans*, was confirmed to be crucial for growth in 0.039% CO_2_ conditions (Bahn et al. 2005). Kim et al. (2010) also noted that when *C. neoformans* infects a human host, its CA plays an important role in CO_2_ induction and metabolism due to the dramatic difference of CO_2_ concentration between the natural environment and the host body. Han et al. (2010) showed that CAs were involved in the pathopoiesis of *Aspergillus fumigatus* and the expression of two kinds of CA of *Aspergillius nidulans* were both relative to CO_2_ concentration. Recently, however, a study indicated that the filamentous ascomycete *Sordaria macrosporacodes* may be able to use traces of HCO_3_^−^ for growth without the participation of CA genes under about 0.039% CO_2_ concentration (Lehneck et al. 2014). For bacteria, *Escherichia coli* encodes two cytosolic CAs, CynT and Can, which have a low identity between protein sequences (Smith and Ferry 2000). The expression of CynT protein is auto‐regulated according to the change in CO_2_ concentration (Guilloton et al. 1992), whereas Can is supposedly involved in the biosynthesis of lipids, amino acids, or nucleotides, and cell growth rate and their density all influenced Can expression (Merlin et al. 2003). These results showed that microbial CAs are presented as having different importance with regard to their life activities in various CO_2_ concentrations.

Insoluble minerals are always sources of inorganic nutrients for the life on rocks. The H^+^ produced by microbial CA catalysis of CO_2_ hydration can promote mineral dissolution and thus allow microorganisms to have easier access to mineral nutrition. This process is beneficial for the growth of the microorganism and promotes transformation of atmospheric CO_2_ (typically at 0.039% concentration). The CA secreted by microorganisms accelerates calcium dissolution from limestone, benefiting their growth (Li et al. 2007). The extracellular CA secreted by *Pseudomonas fragi* is crucial to HCO_3_^–^ transportation in soils rich in calcium carbonate (Sharma et al. 2009). CAs determine whether or not the organisms are capable of living in harsh environments (Kaur et al. 2012). Xiao et al. (2012) found that *A. fumigatus* accelerated potassium feldspar (K‐feldspar) dissolution by improving CA gene expression. Sun et al. (2013) have further shown that CA gene expression by *Aspergillus niger* increased weathering of potassium‐bearing rock. Moreover, the increased expression of CA by *Bacillus mucilaginosus* favors its survival when the growth environment lacks Ca^2+^, but is rich in calcite (Xiao et al. 2014). These results suggested that microbial CAs are related to mineral nutrition acquisition. However, whether microbial CA can really promote mineral dissolution remains a pressing question. Furthermore, the aforementioned *A. fumigatus* and *A. niger* both contain two different CA genes. What is the function of the other?

Although different reports show that CAs are related to acquisition of mineral nutrition or CO_2_ concentration, the presence of multiple CA genes in the same species suggest that the specific functions of one kind of CA gene may be different. However, there is no report available as to whether different CAs in the same species are related to mineral weathering or CO_2_ concentration, respectively. However, the aforementioned concept is important for studying co‐evolution of the Earth's surface minerals, organisms, and biogeochemical cycles. In the work presented here, we use real‐time quantitative PCR (RT‐qPCR) and/or northern blot to study the effect of sufficiency or deficiency in potassium and CO_2_ concentrations on CA gene expression. Moreover, the function of some CA in mineral dissolution was investigated using heterologous expression and protein purification. The object of the study was to explore whether the selective expression of CA genes of *A. nidulans* in response to changes in mineral nutrition and CO_2_ concentration exists.

## Materials and Methods

### Minerals

K‐feldspar was collected from Fuquan in Guizhou Province. The mineral (in powder form) was collected by sieving (100–200 mesh) and the crushed raw material was thrice‐washed with ultrapure water followed by an absolute ethanol wash to remove dust adsorbed on the particle surfaces due to electrostatic interaction. After being washed, they were oven‐dried overnight. Analysis using X‐ray diffraction (XRD) showed that the K‐feldspar mineral powder contained about 78.39% K‐feldspar and 12.18% mica, plus minor chlorite, montmorillonite, and hornblende contents: X‐ray fluorescence (XRF) showed that this powder contained 9.47% K_2_O, 54.55% SiO_2_, 17.52% Al_2_O_3_, plus traces of Fe, Mg, Ca, and Na.

Biotite powders, from Qinghai Yingda Mining Industry Company, contained more than 95% biotite. Wollastonite particles were supplied by the Institute of Geochemistry, Chinese Academy of Sciences. The mineral composition of wollastonite was more than 95% Ca_3_(Si_3_O_9_), and the remainder was mainly quartz. These two kinds of mineral powders used here were treated as described above for the K‐feldspar powders.

### Experimental strain selection

The strain *A. nidulans* TN02A7 (Nayak et al. 2006) was chosen for this study. The cDNA lengths of the two CAs are 699 bp (GI:67538882, CA1 gene) and 923 bp (GI:259487151, CA2 gene), respectively.

### Strain culturing

In this study, we used the MMPDRUU medium as is mentioned in previous reports (Wang et al. 2012). Briefly, MMPDR is a minimal medium with 2% glucose, nitrate salts, trace elements, 0.5 mg L^−1^ pyridoxine, 2.5 mg L^−1^ riboflavin (/L: 20 × salt, 50 mL; trace elements, 1 mL; and water up to 1 L. Among which, salt solution (/L): NaNO_3_ 60 g, NaH_2_PO_4_ 15.2 g, MgSO_4_·7H_2_O 5.2 g; trace elements (/L): ZnSO_4_·7H_2_O 22 g, H_3_BO_3_ 11 g, MnCl_2_·4H_2_O 5 g, FeSO_4_·7H_2_O 5 g, CoCl_2_·5H_2_O 1.6 g, CuSO_4_·5H_2_O 1.6 g, (NH_4_)_6_Mo_7_O_24_·4H_2_O 1.1 g, EDTA 50 g); MMPDRUU, MMPDR with 1.2 g L^−1^ uridine and 1.1 g L^−1^ uracil. MMPDRUU contained different potassium sources, and one medium saw the addition of 0.52 g/L KCl and a second, some 5.2 g/L K‐feldspar powder. The pH values of the liquid media were adjusted to 6.58. Potassium source and CO_2_ concentration were the two variable factors used for cultivation of *A. nidulans*. A 1 mL aliquot of suspended spore liquid (~2.0 × 10^7^/mL) was inoculated into 100 mL of culture medium. The culture conditions were set at 37°C and 220 rpm at 0.039% CO_2_ concentration in accordance with earlier reports (Wang et al. 2012). The pH value of the culture solution was tested at set sampling times (24, 36, and 48 h) using a pH‐meter (METTLER‐TOLEDO SevenEasy S20, Switzerland). The dissolved oxygen was determined with a multifunction water quality analyzer (WTW Multi 350i, Munich, Germany). For experiments involving high‐CO_2_ conditions, the culture environment was bubbled with CO_2_ and the concentration was set to 3.9%.

### Northern blot analysis of the quantity of CA1 mRNA

*A. nidulans* was cultured in the presence of different potassium sources at 0.039% CO_2_ concentration. The differential expression of CA1 was subsequently investigated at 24 h sampling point through a northern blot test. Three independent experiments were carried out in both experimental treatments (with KCl or K‐feldspar as the potassium resource). Digoxigenin (DIG)‐labeled primers (DIG‐High Prime mix, Roche, Switzerland) were used in this work: 5 *μ*g of total RNA was separated onto a 1.2% agarose‐formaldehyde gel and transferred onto a Hybond membrane (Pharmacia, Sweden). The blots were hybridized at 50°C and washed at 68°C with DIG‐labeled DNA probes according to the kit protocol (DIG Northern Kit; Roche). The following manipulations were carried out according to the manufacturer's instruction manual.

### Construction of CA1 expression vector, CA1 preparation, and its effect on mineral dissolution

An *E. coli* expression system, a very common and effective protein expression system (Sørensen and Mortensen 2005), was used for the expression of the CA1 gene in this study. With cDNA as a template, amplification of CA1 produced a sequence containing Kpn1 and EcoR1 restriction sites. A pET30a vector with a strong T7 promoter was introduced to construct the plasmid of CA1 for heterologous expression. Recombinant plasmids were transferred into *E. coli* BL21 to express *N*‐terminal 6 histidine‐tagged protein. After inducible expression using isopropyl *β*‐d‐thiogalactopyranoside (IPTG) was completed, thalli were collected (16,200 g, 4°C, 10 min). They were subsequently re‐suspended using lysate (50 mmol NaH_2_PO_4_, 300 mmol NaCl, 10 mmol imidazole, pH 8.0), and finally broken up using ultrasonication. The crude enzyme solution obtained was centrifuged (16,200 g, 4°C, 20 min). Supernatant protein was collected and purified using Ni‐NTA agarose (Kim et al. 2012). Then, the eluent containing the target protein was dialyzed in dialysate (100 mmol tris‐sulfate 100, pH 8.0) two times (16 h in total). The whole process was carried out at 4°C. SDS‐PAGE (12.5% polyacrylamide) was used to analyze the target protein as described by Laemmli (1970) with some modifications. The protein concentration was measured with bovine serum albumin (BSA) as the standard protein.

The mineral (0.1 g, K‐feldspar/biotite/wollastonite) was separately added to an Erlenmeyer flask. At least three replicates were made. Ultrapure water (49 mL) was quickly placed into the Erlenmeyer flask. Then, 1 mL of sterilized ultrapure water, or the same amount of protein (1 mL), was rapidly added to the flasks, respectively. The flasks were held at 35°C and 130 rpm for 20 min. Thereafter, the liquids were collected and filtered using a 0.45 *μ*m filter membrane. Soluble ion concentration was then measured using ICP‐AES. A two‐tailed *t*‐test was used with STATISTICA 6.0. The data met the assumptions of the test. Significant difference was at 0.05 level. The mean values (along with the standard deviation) were calculated based on three independent experiments.

### Real‐time quantitative PCR to test the expression of CA genes

In our experiment, RT‐qPCR was used to investigate whether or not the CA genes of *A. nidulans* responded to changes in CO_2_ concentration and mineral nutrient availability. Three replicates were made. Mycelium were collected and rapidly frozen in liquid nitrogen. Then, RNA was extracted (following the Qiagen specification, Germany) and reverse transcribed into cDNA. We used 18S RNA (forward primer: CGGCTACCACATCCAAGGAA, reverse primer: GCTGGAATTACCGCGGCT) as an internal reference. The primers for CA1 (forward: AGAAGAGTGTTGTGGTTGAG, reverse: GTCAGAAGCACTGGTATCC) and CA2 (forward: GCTCCATGTCCGTCATC, reverse: TATGCCCAGGTCTGTAGG) were given. The reaction system and conditions used for RT‐qPCR were all taken with reference to the manufacturer's instructions (SYBR^®^ Premix Ex Taq^™^ (TliRNaseH Plus), TaKaRa, Japan). The dissociation curve of RT‐qPCR showed that the PCR products amplified by using suitable primers for the two CA genes had a single melting temperature. The *Ct* value was recorded for subsequent analysis. When the fluorescent signal of each reaction tube reached a set threshold, the number of reaction cycles was the *Ct* value. The *Ct* value of 18S rDNA was found to differ across different concentrations of template, such as N1 and N2. The *Ct* values of targeted genes were recorded, such as M1 and M2. The relative expression level (REL) of targeted genes was _∆_ *Ct* (M1 – N1 or M2 – N2). If we wanted to test the differentially expressed targeted genes with different treatments, the relative expression, _∆∆_ *Ct*, was obtained by subtracting _∆_ *Ct* from two tested genes {(M1 – N1) – (M2 – N2)}. The difference of one cycle meant a change in twofold expression quantity; therefore the REL was calculated thus: *REL* = 2^−*ΔΔCt*^.

## Results

### CA1 gene expression and its involvement in K‐feldspar weathering

**Figure 1:**
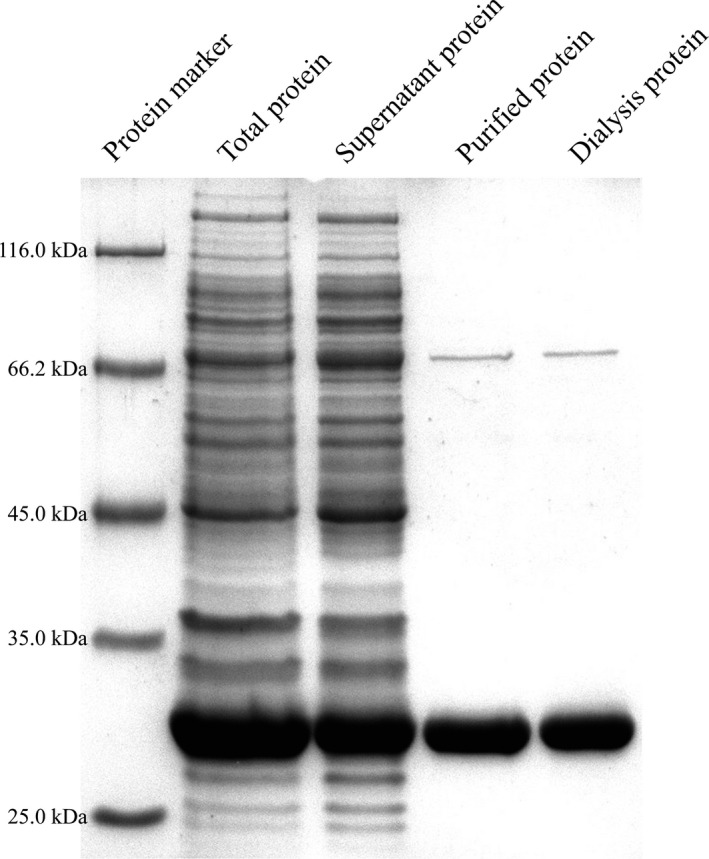
SDS‐PAGE analysis of recombination CA1.

The SDS‐PAGE results (Fig. 1) showed that the protein size was about 27.5 kDa, in agreement with the calculated value (27.24 kDa). The protein concentration was measured about 0.63 mg/mL. The purified protein contained traces of protein impurity at about 63 kDa. This may have been due to the expression of a histidine‐tagged protein by the expression system itself. Another reason may be this protein binding to CA.

For the effect of CA1 protein on K‐feldspar weathering, the results showed that the average K^+^ concentration was almost equal to that of the group without added proteins (Table 1). Biotite and wollastonite, as types of bedded and catenate silicate, respectively, can be used for further exploring whether CA1 was involved in silicate weathering. The amount of K dissolved‐out in the reaction system containing CA1 (0.63 ± 0.09 mg/L) was several times that of the group without added proteins (0.28 ± 0.06 mg/L). CA1 also promoted the dissolution of wollastonite. The concentrations of Ca and Si were increased two to threefold after adding CA1. This verified that CA1 of *A. nidulans* exhibited an auxiliary effect in the process of fungal weathering of minerals.

**Table 1:** Ion releasing from three kinds of minerals with or without CA1

Mineral	Elements	Ion concentration (mg/L)^a^	
		Without CA	With CA
K‐feldspar	K	0.35 ± 0.02	0.37 ± 0.05
Biotite	K	0.28 ± 0.06	0.63 ± 0.09
Wollastonite	Ca	5.71 ± 0.26	9.51 ± 0.47
	Si	1.61 ± 0.013	4.93 ± 0.07

a Data shown were the mean (along with the standard deviation) of at least three independent experiments.

**Figure 2:**
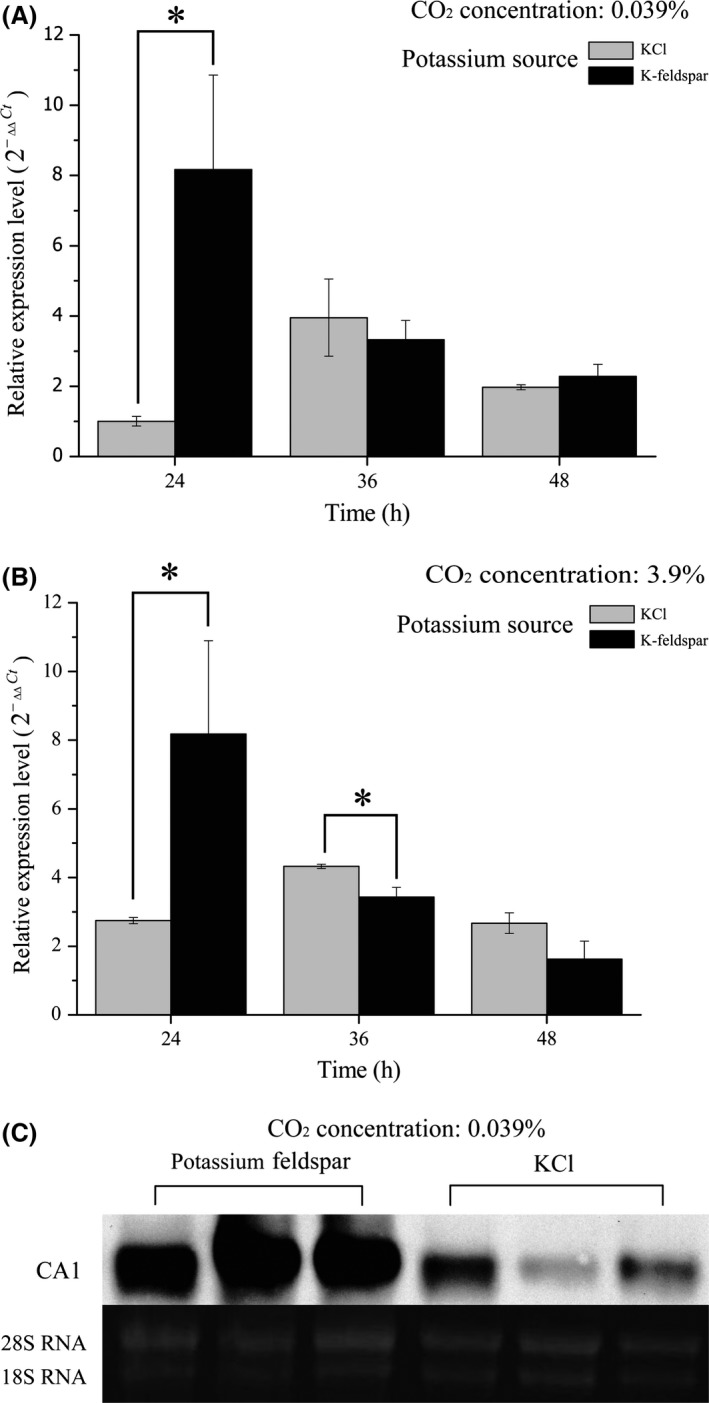
Gene expression of *A. nidulans* CA1 at different temporal sampling points. (A) the expression of CA1 gene with KCl or K‐feldspar at ambient atmosphere (0.039%); (B) CA1 gene expression with KCl or K‐feldspar at high CO_2_ concentration (3.9%). (C) Northern blot analyses of CA1 gene transcription at the 24 h sampling point for *A. nidulans* cultured in K‐feldspar or KCl containing media. Two‐tailed *t*‐test was used. Data shown were the mean (along with the standard deviation) of three independent experiments. *The results from the two treatments are significantly different.

**Figure 3:**
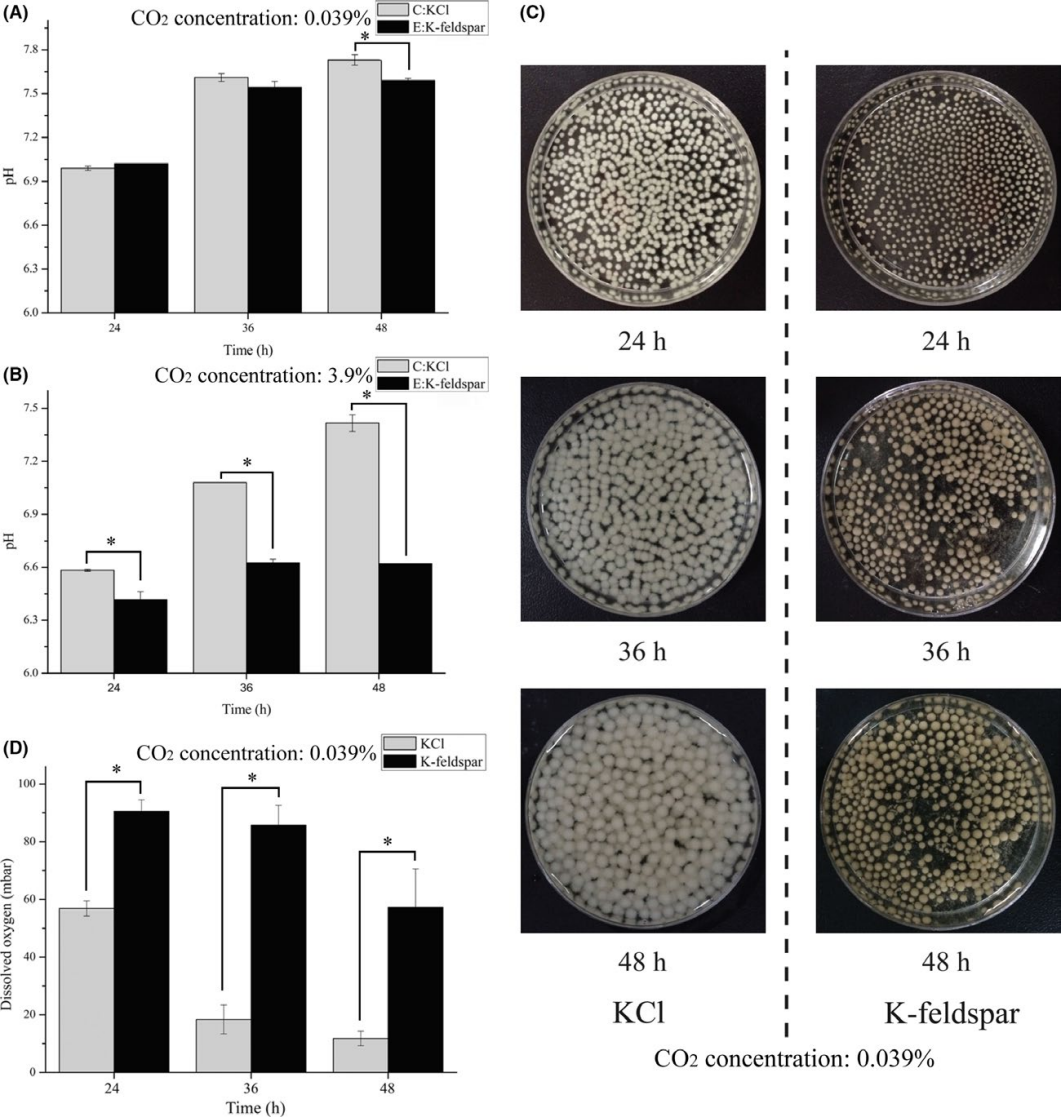
pH values and dissolved oxygen in the medium and appearance of mycelium pellets at three sampling time. (A) pH values of the medium, *A. nidulans* was cultured with KCl or K‐feldspar, named as C or E, at ambient CO_2_ concentration; (B) C and E represent the pH values of medium containing KCl or K‐feldspar, respectively, at 3.9% CO_2_ concentration; (C) size and color of mycelium pellets at three sampling time; (D) the dissolved oxygen of medium at three sampling time. *The results from the two treatments are significantly different.

When *A. nidulans* was cultured using different potassium sources (KCl and K‐feldspar) at 0.039% CO_2_ concentration, CA1 was significantly differentially expressed (*P *=* *0.044) after 24 h (Fig. 2A). The expression level using K‐feldspar was clearly many times (or even an order of magnitude) larger than KCl. The difference was not obvious, however, at both 36 (*P *=* *0.542) and 48 h (*P *=* *0.338). With KCl as the potassium source, REL gradually increased at 36 h and decreased at 48 h (see Fig. 2A). Comparing A and B in Figure 2, the trends observed with 3.9% CO_2_ (Fig. 2B) were similar to those with 0.039% CO_2_ (Fig. 2A). Northern blot results showed that the amount of RNA was high in all three repeats when the potassium source was K‐feldspar (Fig. 2C) verifying that CA1 was required for K‐feldspar weathering. In terms of pH value, both treatments gradually rose at 0.039% CO_2_ concentration (Fig. 3A). There was no statistical difference at 24 and 36 h: although it was different at 48 h, the difference was not large.

### CA2 gene expression for adapting to change in CO_2_ concentration

**Figure 4:**
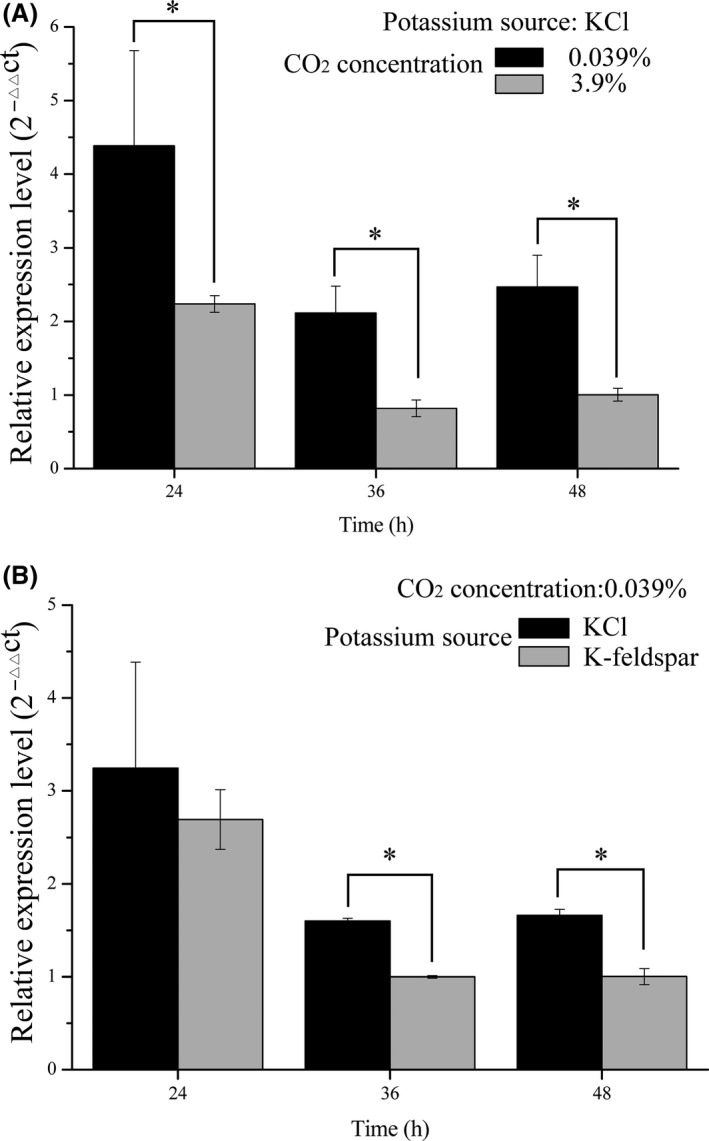
CA2 gene expression at different sampling times. (A) The expression of CA2 gene with KCl as the potassium resource at 0.039% or 3.9% CO_2_ concentration; (B) the expression of CA2 gene with KCl or K‐feldspar as the potassium resource at ambient atmosphere (0.039%). Two‐tailed *t*‐test was used. The mean (along with the standard deviation) were calculated based on three independent experiments. *The results from the two treatments are significantly different.

The results showed that CA2 gene expression was related to the CO_2_ concentration (Fig. 4). When *A. nidulans* was cultured using KCl, the CA2 gene expression was significantly different (*P *≈* *0) at the different CO_2_ concentrations (Fig. 4A). The REL of CA2 showed its highest expression level at 24 h, the expression was negative to CO_2_ concentration. The expression of CA2 gene was irrelevant to potassium sources at 0.039% CO_2_ concentration at 24 h (Fig. 4B). Compared with K‐feldspar, it was higher at 36 and 48 h when KCl was used (see Fig. 4B), and the *P* values were 0.001 and 0.013, respectively. In terms of pH value (Fig. 3B), it was slightly lowered under 3.9% CO_2_ concentration than 0.039% when the potassium resource was KCl. The pH gradually increased with KCl, and the two treatments were statistically significant at any sampling point.

## Discussion

For separate mineral dissolution experiments in this research, the reaction system did not involve microbes, and the only difference was whether the purified protein participated or not. Thus, the effects arising from microbes were nonexistent, such as oxidation and sorption and/or precipitation of ions onto cell biomass. Our recent experimental results showed that the net release of Ca^2+^ from calcite was mainly in the first 20 min when CA was involved (Xiao et al. 2014) and it can also promote the dissolution of wollastonite (Xiao et al. 2015). Thence, ion concentrations were also tested in the first 20 min in this study. The effect of CA1 protein on K‐feldspar dissolution was weak. The main reason may be that K‐feldspar has a framework typical of silicate minerals, so is quite stable. The firmness of K‐feldspar is much higher than the chain structure of wollastonite or bedded biotite. CA1 protein had a significant role in promoting the dissolution of biotite and wollastonite (Table 1). This supplied evidence that CA1 was involved in mineral weathering. The solid phase of these minerals was tested with XRD, and it was not changed by CA dissolution experiments (Fig. S1). It suggested that a single force (catalysis of CA) was not sufficient to make the mineral solid phase change in a relatively short period of time.

Molecular clock estimation indicated that fungi first came into being about one billion years ago (Heckman et al. 2001), and the CO_2_ concentration at that time was 0.001–0.1 bar (Kasting 1987). In this study, CO_2_ concentrations were chosen to be within this range, at 100 times the current atmospheric CO_2_ concentration, and this was adopted as high‐concentration environment (3.9%). No matter what the CO_2_ concentration, the expression of CA1 showed significant differences when *A. nidulans* was cultured with different potassium sources. This provided more evidence that CA1 was involved in mineral weathering. Expression of CA1 gene was at its lowest at 24 h with KCl, indicating that *A. nidulans* did not need to synthesize excessive CA1 protein. However, the soluble potassium content in the K‐feldspar‐bearing culture medium was low in the initial culturing stage. It was clearly insufficient to meet the demand of *A. nidulans* for K^+^ for growth. To obtain enough K^+^, *A. nidulans* needed to destroy K‐feldspar by changing its metabolic pathway. As culturing continued, the amount of CA1 protein secreted by *A. nidulans* may be sufficient to meet demand. Liu et al. showed that CA can accelerate mineral dissolution by several times and even up to an order of magnitude (Liu and Dreybrod 1997; Liu 2001), and thus the expression level of the CA1 gene gradually decreased to avoid meaningless expression (Fig. 2A). Prolonged incubation caused the pH value to gradually increase (Fig. 3A). The continuous consumption of H^+^ and/or production of (bi)carbonate may have caused this. Note that a slight reduction in pH may be expected at 3.9% CO_2_ concentration at 24 h due to CO_2_ dissolving into the medium (Fig. 3B). More H^+^ attacked the K‐feldspar and caused K^+^ release. The demand for soluble potassium was relatively low, so CA1 expression gap became narrower at 24 h (comparing Fig. 2A and B). In addition, the result from a northern blot test (Fig. 2C) further verified the positive participation of CA1 in K‐feldspar dissolution.

When *A. nidulans* was cultured at different CO_2_ partial pressures, CA2 gene expression changed significantly (Fig. 4A). CA2 gene expression was much lower at 3.9% CO_2_ concentration. Previous research (Forkman and Laurell 1966; Fukuzawa et al. 1990; Hashimoto and Kato 2003) has shown that CA presented lower expression levels in higher CO_2_ environments. It is still unclear if there was a threshold CO_2_ concentration affecting CA2 expression. For example, when the CO_2_ concentration was lower than this threshold, over‐expression of CA2 arose. Conversely, when the CO_2_ concentration exceeded this threshold, CA2 expression was likely to be reduced. The REL of CA2 gene was not significantly different at 24 h under a 0.039% CO_2_ concentration for the different K sources (Fig. 4B). It was plausible that CA2 participation was not required for mineral weathering since CA1 played a key role in this regard. Regardless of this, at 36 h and 48 h, the REL of the CA2 gene was significantly different (*P *=* *0.010 and *P *=* *0.011, respectively) (Fig. 4B). In contrast with CA1, the expression level of CA2 was higher with KCl than K‐feldspar. This phenomenon may correlate with the growth environment of *A. nidulans*. In this experiment, the mineral particles were wrapped by mycelium pellets and were faint yellow in color in Group E (K‐feldspar as the K source) (Fig. 3C). This was in contrast to the milk white appearance in the Group C (KCl as the K source). The sizes of the mycelium pellets were much larger in Group C. Furthermore, the number of mycelium pellets in Group C (1014 ± 30) was greater than in Group E (754 ± 90). Some research (Puskás et al. 2000; Kaluz et al. 2002) showed that microbial CA was expressed in anaerobic environments but, in aerobic environments, CA expression was lower. Since the biomass of Group C was higher than that of the Group E, more O_2_ was thus consumed (Fig. 3D): similar behavior with CA2 may be partly due to consumption of O_2_.

To sum up, this study showed that one of the two CA genes was a preference gene when *A. nidulans* grew under special conditions. CA1 was involved mainly with the acquisition of mineral elements, while CA2 mainly adapted to changes in CO_2_ concentration. The directional selection of the *A. nidulans* CA isozyme could ease some of the adverse effects brought about by external changes in the environment. It could also help *A. nidulans* avoid the waste of materials and energy caused by the genetic overexpression. Inversely, their adaption may impact mineral weathering and/or CO_2_ concentration, and even global change.

## Ethical Statement

This article does not contain any studies with human participants or animals performed by any of the authors.

## Conflict of Interest

None declared.

## Supporting information


**Figure S1.** XRD analysis of the solid phase of three types of minerals before and after CA dissolution experiments. a, c and e represents three types of minerals before CA dissolution experiments. b,d and f represent three types of minerals after CA dissolution experiments.
