# Supplementary figures and images for: The selective expression of carbonic anhydrase genes of Aspergillus nidulans in response to changes in mineral nutrition and CO 2 concentration
Source: Microbiologyopen. 2015 Nov 9;5(1):60–9. doi: 10.1002/mbo3.311 (PMC4767425; doi:10.1002/mbo3.311)

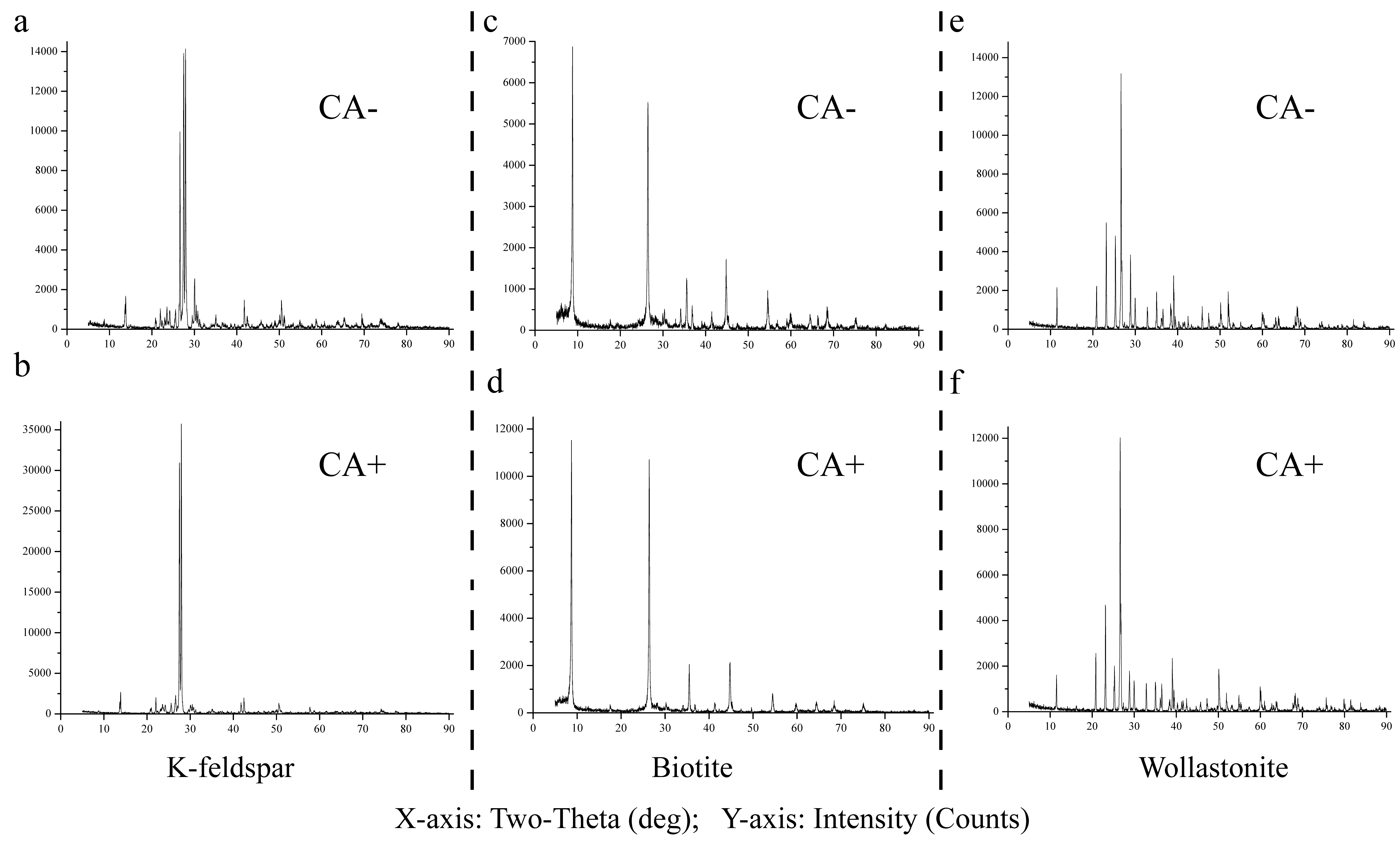

Supplement: Supplementary file 1 — Figure S1. XRD analysis of the solid phase of three types of minerals before and after CA dissolution experiments. a, c and e represents three types of minerals before CA dissolution experiments. b,d and f represent three types of minerals after CA dissolution experiments. [file MBO3-5-060-s001.tif]
